# Cohen’s MRI scoring system has limited value in predicting return to play

**DOI:** 10.1007/s00167-016-4403-8

**Published:** 2017-02-04

**Authors:** Bruce Hamilton, Arnlaug Wangensteen, Rod Whiteley, Emad Almusa, Liesel Geertsema, Stephen Targett, Johannes L. Tol

**Affiliations:** 1 Aspetar, Orthopaedic and Sports Medicine Hospital, Doha, Qatar; 2 High Performance Sport NZ, Sport Research Institute of New Zealand, Millenium Institute of Sport and Health, Mairangi Bay, Auckland, New Zealand; 3 Oslo Sports Trauma Research Center, Department of Sports Medicine, Norwegian School of Sports Science, Oslo, Norway; 4 Acedemic Center for Evidence Based Medicine (ACES), Academic Medical Center, University of Amsterdam, Amsterdam, The Netherlands

**Keywords:** Muscle injury, MRI, Return to play

## Abstract

**Purpose:**

Numerous authors have hypothesised that MRI scoring systems provide a valid means of predicting return to play duration following an acute hamstring muscle strain. The purpose is to prospectively investigate the predictive value of the MRI scoring system of Cohen for return to sport (RTS), following an acute hamstring injury.

**Methods:**

Male football (soccer) players (*n* = 139) with acute onset posterior thigh pain underwent standardised clinical and MRI examinations within 5 days after injury. All players underwent a standardised physiotherapy regimen with RTS documented. The MRI scoring was statistically evaluated against RTS.

**Results:**

One hundred and ten MRI-positive hamstring injuries were evaluated with RTS duration ranging from 1 to 66 days. Total Cohen’s MRI score accounted for approximately 4% of the variance in RTS duration. When comparing those with an MRI score of 10 or more took on average 9.8 days longer to RTS than those with an MRI score less than 10 (effect size: 0.85, *p* < 0.01).

**Conclusions:**

Utilising the Cohen’s MRI scoring system previously described, we were unable to provide a clinically useful prognosis for RTS in male soccer players. This may reflect the broader challenges of attempting to accurately determine RTS duration from imaging performed at a single point in time.

**Level of evidence:**

Prospective case series, IV.

## Introduction

Hamstring muscle injuries remain one of the most common injuries in sport, with a correspondingly high re-injury rate [10–13, 19]. For medical staff, it remains a challenge to accurately diagnose and treat hamstring muscle injury. Furthermore, providing an accurate estimation of return to sport (RTS) proves to be even more difficult.

Recently, a number of authors have presented classification and grading systems for muscle injuries, with the goal of assisting in the prediction of RTS duration [4, 5, 21, 24]. Ekstrand et al. and Mueller-Wohlfhart et al. [10, 21] presented a comprehensive system for the classification and grading of muscle injuries, incorporating history, examination and radiological features, which was subsequently validated suggesting some predictive capability in footballers. Despite conflicting evidence [20, 25], authors have attempted to grade and classify hamstring injuries based solely on MRI interpretation, in an effort to improve prediction of time to RTS. Pollock et al. [23, 24] described a system for the grading of muscle injuries in track and field competitors, and a retrospective validation supports elements of its classification. Chan et al. [4] described an MRI-based classification and grading methodology but present no data to support this approach.

Cohen et al. [5] developed an MRI-based grading system, incorporating a novel scoring system based on age and a range of MRI variables. In a retrospective analysis of 43 injuries in NFL players over a 10-year period, Cohen et al. assessed the relationship between their MRI observations and the number of games lost to injury. The authors concluded that a rapid return to play was more likely in those injuries with an MRI score of less than 10, compared to a score of greater than 10 (range 11–maximal 16). Indicators of a poor prognosis included multiple muscle involvement, a higher percentage (>75%) of transverse muscle involvement, more than 10 cm of craniocaudal involvement and muscle retraction. Ultimately, they concluded that their MRI score was useful in determining injury severity and predicting RTS duration in professional footballers.

However, the work of Cohen has several limitations, including its retrospective nature with potential recall bias, limited subject numbers and a lack of detail regarding the return to sport process. Our goal was to prospectively evaluate the predictive value of Cohen’s MRI scoring system in a football (soccer) setting.

## Materials and methods

This study is based on pooled data from a randomised controlled trial (RCT) [15] and a prospective cohort study of acute hamstring injuries [28]. Both studies were conducted at Aspetar Hospital, Qatar. Written informed consent was obtained from all patients.

Eligibility criteria for enrolment in both pools of the study are presented in Table 1. Between 2011 and 2014, professional football players with MRI-positive hamstring strain injuries were recruited from sporting clubs and federations in Qatar. All patients were assessed by a sports physician using a standardised approach [15], including an MRI within 5 days of injury.

**Table 1:** Study eligibility criteria

Prospective case series	Randomised controlled trial
*Inclusion criteria* Male professional soccer player Age 18–50 years Acute onset of posterior thigh pain when training or competing ≤5 days after injury Clinical diagnosis ≤5 days after injury MRI performed ≤5 days from injury Available for follow-up MRI confirmed hamstring lesion *Exclusion criteria* Re-injury ≤2 months after RTS [11] Chronic hamstring complaints >2 months Grade III hamstring tear Contraindications to MRI Already included with prior injury	*Inclusion criteria* Male professional soccer player Age 18–50 years Acute onset of posterior thigh pain Presenting and MRI within 5 days from injury MRI confirmed hamstring lesion Able to perform five sessions of physiotherapy a week at our clinic Available for follow-up *Exclusion criteria* Contraindication to MRI Re-injury ≤2 months after RTS [2] or chronic hamstring injury >2 months Other concurrent injury inhibiting rehabilitation Unwilling to comply with follow-up Needle phobia Overlying skin infection Diabetes, immune-compromised state Medication with increasing bleeding risk Medical contraindication to injection

Athletes in the RCT study were randomised into three groups, receiving an injection of platelet-rich plasma (PRP), platelet-poor plasma (PPP) or no injection. Each group followed a 6-stage criteria-based physiotherapy programme including sports-specific functional field testing designed to mimic the fatigue and competitiveness of full unrestricted training [27]. The RCT showed no benefit of PRP or PPP over no injection but a delayed time to RTS for PPP compared to PRP. The athletes included in the prospective case series received either the rehabilitation described above or a custom-made rehabilitation at either the study centre or their club or federation.

Time to RTS was defined as the number of days from initial injury until the athlete was cleared to resume full, unrestricted training by the treating physician. Players receiving rehabilitation at the study centre were evaluated by the treating physician on the day of completing the final stage of the functional field testing. Criteria for the RTS decision-making included successful and asymptomatic completion of the criteria-driven rehabilitation programme, clinical evaluation and the subjective interpretation of an isokinetic assessment [27]. For athletes receiving rehabilitation in the clubs or federations, time to RTS was recorded as when the athlete returned to full, unrestricted training. The time to RTS was provided by club medical staff to the research team by phone or email on a weekly basis. The RTS decision-makers were not blinded to the baseline assessments or the MRI findings, but were blind to the Cohen MRI score, which was not included in the MRI report.

### Magnetic resonance imaging

MRI images were taken of the hamstring muscles within 5 days of injury with a 1.5-T magnet system (Magnetom Espree, Siemens) using a body matrix coil. Initially, coronal and transversal proton density (PD)-weighted images (TR/TE of 3000/30 ms, FOV of 220–240 mm, slice thickness of 5 mm and a 333 × 512 matrix) were collected. Subsequently, coronal and transversal proton density fat saturation (PD-FS) images (TR/TE of 3000+/30 ms, FOV of 220–320 mm, slice thickness of 3,5 mm, a 326 × 512 matrix for the coronal images and a 333 × 512 matrix for the transversal images) were obtained.

MRIs positive for injury were scored according to the variables as described by Cohen et al. (Tables 2, 3) [4] by a radiologist with more than 9 years of experience in musculoskeletal radiology (EA). Using the same radiologist, excellent inter- and intra-rater correlation coefficients (ICCs) for grading and MRI variables have been previously reported [17].

**Table 2:** Cohen’s MRI descriptors for reporting hamstring muscle injury [5]

MRI variable	Detail
Muscles or tendons involved	Semimembranosus; biceps femoris short head, biceps femoris long head, semitendinosus
Location of involvement	Origin avulsion, proximal myotendinous junction, muscle belly, distal myotendinous junction, insertion avulsion
Cross-sectional area percentage of involvement	0, 25, 50, 75, 100%
Tendon or muscle retraction	Centimetres
Signs of chronic tendinopathy	Abnormal morphology or signal in uninjured structures, peritendinous and perimuscular oedema, intramuscular cysts
Craniocaudal sagittal extent	Extent of abnormal hyperintense signal on the T2 PD-FS-weighted sequences. Centimetres

*PD*-*FS* proton density fat saturated

**Table 3:** Scoring of MRI variables measured (based on Cohen et al. [5])

Score	Age (years)	Muscles involved (*n*)	Location	Insertion	Muscle injury (transverse %)	Retraction (cm)	Sagittal length (cm)
0				No	0–<25	None	0
1	≤25	1	Proximal		25–<50	<2	1–<5
2	26–31	2	Middle	Yes	50–<75	≥2	5–<10
3	≥32	3	Distal		≥75		≥10

*cm* centimetres

The study protocol was approved by the Ethics Committee of Aspetar Orthopaedic and Sports Medicine Hospital and the Anti-Doping Laboratory Qatar Ethics Committee (reference number 2012-018).

### Statistical analysis

Examination of the relationship between the Cohen MRI score and the time to RTS was initially performed using descriptive statistics (including tests of normality) and construction of a simple scatter plot, upon which a line of best fit and associated 95% confidence intervals were constructed. As the test for normality (Kolmogorov–Smirnov) was violated for both variables (significance for Cohen’s MRI score: 0.003 and time to RTS: 0.041), Spearman’s rho was estimated as the correlation coefficient. Finally, the group was split into two cohorts: those with a Cohen MRI score of less than (<10) or greater than/equal to 10 (>10). These two groups were compared for their time to RTS with descriptive summary statistics, between group differences and their associated 95% confidence intervals. No formal sample size calculation was performed.

## Results

We identified 139 professional footballers with a clinically suspected acute hamstring injury. Twenty-eight injuries were MRI negative, and one injury did not have a cross-sectional area documented, and they were therefore excluded from the analysis. Of the 110 included footballers, the average age at injury was 26 years (range 18–39 years), height 177 cm and weight 73 kg and represented a range of ethnicities including 52.7% Arab, 30.0% Black, 1.8% Caucasian, 5.5% South and East Asian, 4.5% Persian and 5.5% other.

In 89 patients (80.2%), only one muscle was identified as injured, in 21 patients (18.9%) two muscles were injured and in one case (0.9%), three muscles were injured. The primary injury was observed to the long head of the biceps femoris (*n* = 89, 81%), semimembranosus (*n* = 17, 15%), semitendinosus (*n* = 3, 3%) or the short head of the biceps femoris (*n* = 1, 1%). Twenty-one (18.9%) injuries were located in the proximal muscle, 46 (41.4%) mid-muscle and 44 (39.6%) distally. No insertional injuries or muscle retraction was noted, as complete (Grade 3) ruptures were exclusion criteria. Cross-sectional muscle involvement was <25% (*n* = 76, 69.7%), 25–49% (*n* = 18, 16.5%), 50–74% (*n* = 10, 9.2%) and ≥75% (*n* = 5, 4.6%). Longitudinal muscle involvement was 1–5 cm (*n* = 16, 14.6%), 6–10 cm (*n* = 37, 33.6%) and >10 cm (*n* = 57, 51.8%). The average total Cohen’s MRI score was 7.9 (range 4–12, SD: 1.72). RTS duration ranged from 1 to 66 days with a mean of 22.65 days (SD: 11.03) (Table 4).

**Table 4:** Summary statistics for Cohen’s score and time to RTS

	Total Cohen’s score	Time to RTS (days)
Mean (95% CI)	7.9 (7.5–8.2)	22.7 (20.6–24.7)
Median (IQR)	8 (2)	21 (13)
SD	1.7	11.025
Minimum	4	1
Maximum	12	66
Pearson’s rho	0.205 (*p* = 0.032)	

*RTS* return to sport, *CI* confidence interval, *IQR* interquartile range, *SD* standard deviation

**Fig. 1:**
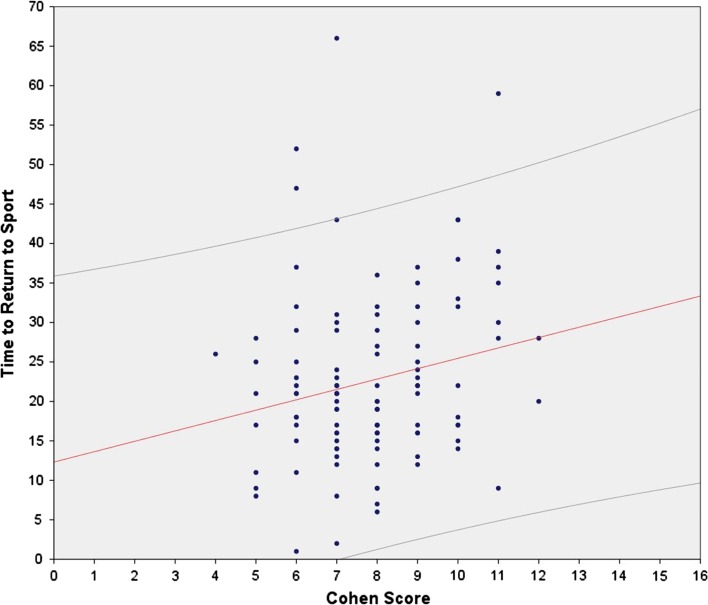
Scatter plot illustrating the correlation between the total Cohen’s score and time to return to sport (days) including regression line (*solid line*) with 95% confidence intervals (*dashed lines*)

**Fig. 2:**
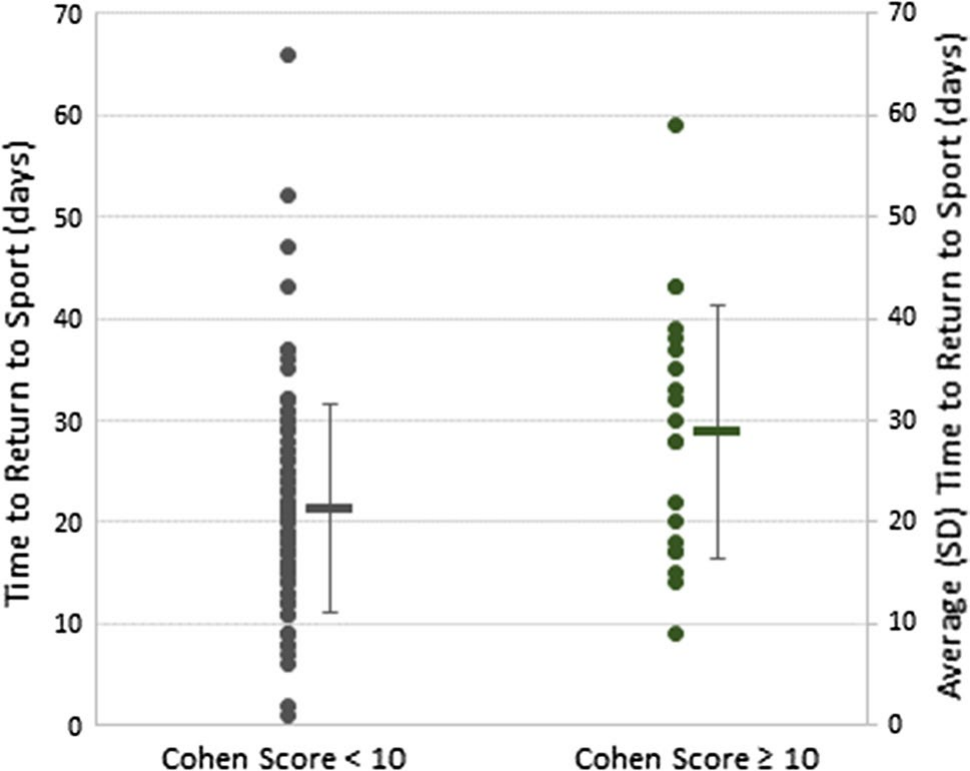
Relationship between total Cohen’s score < 10 and ≥10 with RTS duration. *Dots* represent time to RTS (days; *left axis*) for individual subjects. The *horizontal bars and vertical* whiskers represent mean time to RTS (days) and standard deviation, respectively (*right axis*)

The relationship between the total Cohen’s MRI score and time to RTS is illustrated in Fig. 1. On average, RTS in those with a score of 10 or more (mean 30 days; median 30 days) took 9.8 days longer than a score of less than 10 (mean 20 days; median 19 days; effect size: 0.85, *p* < 0.01) (Fig. 2). Assuming the Cohen MRI score as a continuous variable, Pearson’s correlation coefficient = 0.21 (*p* = 0.03), and when not considered as a continuous variable, Spearman’s correlation coefficient = 0.18 (n.s.), suggesting only approximately 4% of the variance in time to RTS is explained by the Cohen MRI score.

**Fig. 3:**
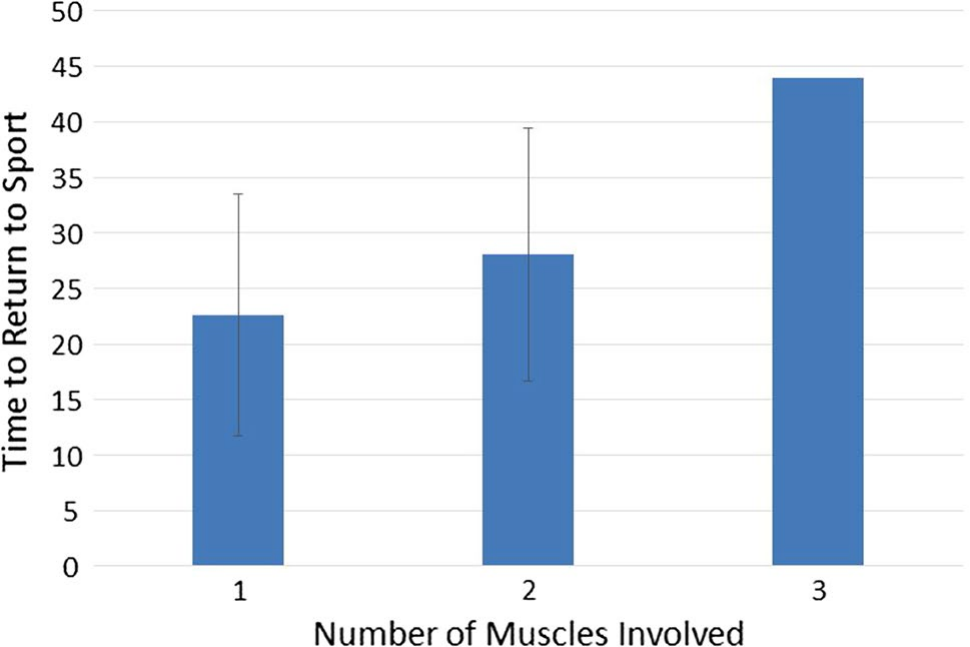
Mean number of days to return to sport grouped by number of muscles involved (whiskers represent SD). Note, as there was only one subject with three muscles involved, no SD is presented for this series

Of the individual variables incorporated into the Cohen model, only the number of muscles involved was found to have a significant relationship with time to RTS (Fig. 3).

## Discussion

The most important finding of this prospective study was that the MRI scoring system of Cohen et al. has no clinical value for predicting time to RTS. Examination of the relationship between the total Cohen’s MRI score and time to RTS shows that nearly all Cohen’s MRI scores identified may be associated with a time to RTS spanning the entire time to RTS range (Fig. 1), and that the Cohen MRI score accounted for only 4% of the variance in RTS duration following an acute hamstring injury.

While a total Cohen’s MRI score of less than 10 had a significantly distinct mean outcome in comparison with those with a score of 10 or more, the large standard deviation effectively eliminates any clinical utility (Fig. 2). Furthermore, of all the variables recorded, only the total number of muscles involved correlated with time to RTS (Fig. 3). Based on this evaluation using the scoring system described by Cohen et al. [5], MRI provided no prognostic relevance for time to RTS. This finding is in conflict with the original retrospective study of 43 hamstring injuries, in which the authors concluded that a higher score, muscle tendon involvement, a high (>75%) muscle involvement and retraction were predictive of a delayed time to RTS, and that MRI was “reliable in determining severity of injury and time away from sport in hamstring injuries…”.

Clinical muscle injury grading systems, purporting to establish injury severity, have been utilised for over 50 years, but until recently have lacked any empirical validation [16]. Since the late 1990s, the use of MRI has become increasingly popular in both elucidating a clear diagnosis and establishing a prognosis, but until recently there has been no attempt to validate their ability to determine time to RTS. The literature remains conflicting on the ability of various grading and classification systems to predict time to RTS [9]. In particular, recent classification and grading systems developed by the “Munich consensus group” [21] and the British Athletics group [23, 24] have proposed comprehensive systems, purporting to provide prognostic value. Despite these classification and grading proposals, recent investigators have questioned the prognostic merit of a single MRI investigation following injury [9, 20]. Rationale for this may relate to the observation that each specific injury pathology will vary in three dimensional size, location, tissue involvement (muscle/tendon/fascia), and will sit within an even more biologically complex individual. Time to RTS following injury depends on a multitude of inter-related factors, of which injury “pathology”, while likely an important factor, sits within a broader psychological, social and political framework [7, 26]. For these reasons, the expectation that a single image may provide an accurate prognosis appears unrealistic [18, 28].

Using the number of games missed as their outcome measure, they found a strong correlation between the total MRI score and outcome, with higher scores missing more games. Using a prospective design, with a more sensitive outcome measure (days missed) and a larger sample, we have been unable to reproduce this finding. There are a number of technical reasons why we found a different result to that of Cohen et al., including difficulties we experienced reproducing the original methodology. Unfortunately, the nature of the original manuscript makes it difficult to determine whether we have followed exactly the same methodology. For example, the muscle injury area is reported as absolute (i.e. 0, 25, 50 or >75%), while we interpreted this slightly differently (see Table 2). Similarly, while the authors report that the lowest MRI score possible is 2, they also describe two MRI-negative injuries (reported as grade 0 on the “traditional MRI grade”); with an MRI-negative injury, it would appear possible to score 1. While Cohen et al. also utilised a “traditional grading system”, we restricted the current assessment to the evaluation of the MRI score developed by Cohen et al., as this is both novel and aligned with other recently proposed MRI grading systems. That Cohen et al. [5] utilised games missed as their outcome measure, while we utilised days missed, may also have altered the findings, although days missed before return to sport, appear a more appropriate measure of injury severity.

Of all the variables measured, only the number of muscles involved was associated with time to RTS. This is in contrast to previous research which has suggested that injury size [14] and location [1, 2] may play a role in time to RTS. Of note, many previous studies assessing the merits of MRI for prediction of time to RTS have typically only assessed the largest or most significantly injured muscle [10, 14]. Our observation that injury size did not correlate with time to RTS, suggests that this approach may be flawed. It is possible that a muscle with a smaller injury, but involving specific elements of the muscle–tendon unit, may be the determining component in time to RTS [3, 6, 8, 24].

Limitations of our study include that the return to sports decision-makers were not blinded to the results of the imaging. Awareness of the imaging may therefore have influenced the time to RTS, although the specific Cohen’s MRI score was not utilised clinically nor were the specific MRI variables reported independently. Furthermore, we have only included MRI-positive injuries, which may not reflect the complete spectrum of hamstring injuries, thereby influencing our RTS duration. Utilising pooled data and differences in setting and rehabilitation received [22] may reduce the accuracy of our findings, although it increases the generalisability. High levels of compliance in reporting data and methodology were generally achieved.

## Conclusions

Using the system of grading hamstring muscle injuries originally described by Cohen et al., which incorporates many factors thought to be relevant in determining the severity of hamstring injuries, we were unable to provide a clinically useful prognosis for return to sport, indicating the lack of clinical relevance of the Cohen classification system.

## Abbreviations

CI: Confidence interval
Cm: Centimetres
IQR: Interquartile range
PD-FS: Proton density fat saturated
RCT: Randomised controlled trial
RTS: Return to sport
SD: Standard deviation
